# Exploring the low-carbon development path of resource-based cities based on scenario simulation

**DOI:** 10.1038/s41598-025-89822-3

**Published:** 2025-02-18

**Authors:** Liyong Cao, Peian Chong

**Affiliations:** 1 School of Architecture and Civil Engineering, Xihua University, Chengdu, 610039 Sichuan China; 2 Shanghai Power Equipment Research Institute Co., Ltd., Shanghai, 200240 China

**Keywords:** Resource-based cities, Resilience, Carbon peaking, Interval forecasting, Scenario simulation, Environmental sciences, Environmental impact

## Abstract

Resource-based cities (RBCs) have historically been constrained by their inherent characteristics, impeding rapid shifts in energy consumption patterns and exerting substantial pressure on regional decarbonization efforts. Herein, 18 RBCs in southwestern China were taken as the research object. Firstly, a resilience index system was constructed for the resource ecosystem and socio-economic system of RBCs, and the optimization mutation level algorithm was used to measure the resilience level of each city. Secondly, an interval prediction model was established for carbon emissions in RBCs based on the GA-DBN-KDE algorithm. Finally, by setting 16 scenarios, the carbon emission range and “carbon peak” time range of RBCs in Southwest China from 2023 to 2040 were predicted, and the scientific path of low-carbon development of RBCs was explored under differentiated scenarios. The research results indicated that: (1) The carbon emissions and urban resilience levels of RBCs in southwestern China were both on the rise; (2) The interval prediction model based on GA-DBN-KDE demonstrated excellent prediction performance; (3) The simulation results of 16 scenarios revealed varying specific paths for 18 cities to achieve carbon peak, underscoring the necessity for city-specific policy formulation. Overall, this paper provides a new analytical method for the low-carbon transformation and development of RBCs, further forging a basis for decision-makers to formulate carbon reduction measures.

## Introduction

*The Paris Agreement*, as a milestone document in global climate governance, clearly sets ambitious goals to control global warming within 1.5 °C or 2 °C, and prescribes the phased tasks to achieve net zero global greenhouse gas emissions by 2050 and 2070, respectively^1^. However, in the process of promoting the “dual carbon” goal, various regions, especially RBCs with high carbon emissions, are facing a profound contradiction between rapid economic growth and sustainable development^2^. RBCs, with their extensive development models of high investment, high energy consumption, high pollution, and low efficiency, have become a major challenge for China to fully achieve its carbon peak goal^3,4^. The concept of urban resilience has been integrated into the four Sustainable Development Goals of the United Nations, including Goal 1.5^5^. In this context, exploring how RBCs can achieve carbon reduction from the perspective of urban resilience is not only related to the sustainable development of each city, but also an important way to provide valuable experience for the global green development path^6^.

To analyze the low-carbon development path of cities from the perspective of urban resilience, the first step is to understand the situation of cities in terms of resilience development. RBCs in southwestern China have invested heavily in enhancing urban resilience, which generally includes ecological resilience and economic resilience levels^7^. Ecologically, projects primarily involve topics such as returning farmland to grassland, returning farmland to forest, windbreak and sand fixation, characteristic forestry and fruit industry, integrated protection and systematic management of mountains, waters, forests, fields, lakes, grasses, and sands^8,9^. The emphasis on ecological protection has led to substantial investment in environmental governance, imposing economic pressures on the resource-based cities in southwestern China. At present, RBCs in southwestern China are still subject to problems such as inadequate resource utilization, low environmental governance efficiency, severe environmental pollution, and unstable industrial structure^10^, seriously restricting the resilience level of RBCs in southwestern China^11,12^.

At present, a multitude of studies employ various methods to quantitatively analyze urban resilience across different dimensions. The specific situation is shown in Table 1:

**Table 1:** Research perspectives and methods on urban resilience.

References	Related indicators	Main methods	Advantages and disadvantages of methods
^13^	Environmental and social benefits	Analytic hierarchy process	Unable to mine data features
^14^	Ecological environment	Entropy weight method	Lack of explanation for weight allocation results
^15^	Society, economy, institutions, ecology, and engineering	Entropy weight—TOPSIS method	Strong demand for data
^16^	Society, economy, infrastructure, environment, and government	System evaluation method	Strong subjectivity
^17^	Society, economy, infrastructure, ecology	CRITIC-EWM method	Ignore the correlation between indicators

Based on the literature review above, among the existing research results, urban resilience is mainly analyzed from the perspectives of ecology, environment, society, and economy, with urban resilience rarely combined with low-carbon development. The quantitative models and algorithms for resilience levels also have certain shortcomings.

The path to low-carbon transition and development for RBCs under the dual carbon framework is garnering growing scholarly interest. Therefore, 18 RBCs in southwestern China were taken as the research object. Based on the analysis of the characteristics of RBCs, a resilience index system for “resource ecology” and "socio-economic" of RBCs was constructed. From the specific indicators affecting urban resilience, the dynamic changes in resilience of RBCs were quantified. A carbon emission interval prediction model was established based on GA-DBN-KDE algorithm, and the carbon emission trend of RBCs was analyzed. Exploring the low-carbon development path of RBCs through scenario simulation, providing scientific basis for the transformation and development of RBCs. The remaining research organization of this paper is as follows: "Literature review" section reviews and summarizes current research literature; "Overview and methods of the research area" section briefly introduces the research framework, methods, and data sources of this paper; "Results" section analyzes the carbon dioxide emissions of 18 RBCs in Southwest China, calculates the resilience levels of 12 dimensions, builds a carbon emission interval prediction model based on GA-DBN-KDE, and sets up 16 differentiated scenarios for interval prediction of carbon dioxide emissions from 2023 to 2040; "Conclusion" section mainly focuses on the results and related policy recommendations; and "Discussion" section presents the discussion.

## Literature review

### Sustainable development theory and the transformation needs of RBCs

Sustainable development theory centers on harmonizing economic growth, social advancement, and environmental conservation to sustainably utilize resources and safeguard the ecological environment for the benefit of current and future generations^18^. For RBCs, the importance of this theory is particularly prominent. These cities have inherently relied on natural resource development to drive economic growth, yet this model is frequently accompanied by problems such as excessive resource consumption, severe environmental pollution, and ecological imbalance^19–22^. Therefore, in order to adapt to the new requirements under the dual carbon goal, it is urgently necessary for RBCs to transform their development model from relying solely on natural resources to a diversified and sustainable industrial structure^23^. In the context of dual carbon, the sustainable development path of RBCs should focus on key areas such as promoting clean energy, improving energy efficiency, and developing circular economy, so as to effectively reduce carbon emissions and promote green economic growth^24^. In addition, strengthening ecological protection and environmental governance, and restoring and improving the quality of the ecological environment, are important guarantees for RBCs to achieve sustainable development^25^. Notably, the United Nations’ Sustainable Development Goals have explicitly incorporated “urban resilience” as a key element^5^, emphasizing the ability of cities to resist, adapt, and recover from external shocks, which is highly in line with the needs of RBCs transformation.

### Urban resilience theory and resilience assessment of RBCs

The urban resilience theory has gradually constructed a complete system, encompassing theoretical exploration, indicator development, quantitative analysis, and resilience enhancement strategies^26^. However, the existing research on the differential analysis of RBCs as a specific object is still insufficient. Grasp of the “resilience” concept is fundamental to assessing urban resilience, which is generally understood as a system’s capacity to sustain functionality, reorganize, and recover amidst change and disruption^27^. As a special group of cities, the resilience assessment of RBCs should pay special attention to the challenges of resource and ecological dimensions (such as resource consumption, resource reserves, ecological environment pollution, and ecological balance) as well as socio-economic dimensions (such as insufficient follow-up power and imbalanced industrial structure)^28,29^. In the context of dual carbon, analyzing the resilience level of RBCs in these key dimensions is crucial for formulating effective resilience enhancement strategies. This not only assists RBCs in better coping with the dual pressures of external environment and internal development, but also is a key step in achieving green transformation and promoting sustainable development.

### The theory of urban transformation and the green development path of RBCs

The urban transformation theory focuses on how cities can achieve transformation, upgrading, and sustainable development through industrial structure adjustment, spatial layout optimization, and urban function enhancement when facing external environmental changes or internal development pressures^19^. For RBCs, this theory provides important guidance. Faced with development bottlenecks such as resource depletion, environmental pollution, and a single industrial structure, RBCs must transform, achieve industrial diversification and upgrading, and promote sustained and healthy economic development. Under the dual carbon initiative, RBCs should prioritize green and low-carbon development in their transformation efforts^30^. By vigorously promoting clean energy, deepening the development of circular economy, and strengthening technological innovation, the transformation of industrial structure towards green, low-carbon, and efficient direction will be promoted. At the same time, efforts should be made to optimize urban spatial layout, enhance urban functions, and strengthen the overall competitiveness and low-carbon development capacity of the city^31^. This process not only facilitates RBCs to break free from excessive dependence on natural resources, but also provides the possibility for achieving green economic growth and improving the ecological environment.

At present, there are many studies on the path of green development in RBCs^32,33^. RBCs in the United States mainly promote sustainable development through measures such as building and transportation emissions reduction. By establishing a three-level system of "vision strategy indicators", focusing on buildings and transportation as key areas for emission reduction, the objective is to achieve zero emissions from buildings, green building design, and green transportation^33^. The green development of RBCs in the UK is mainly achieved through supplementary planning guidelines such as the "Guidelines for Carbon Assessment throughout the Life Cycle", which provide detailed guidance and implementation of corresponding policies, offer feedback on the implementation effect of the plan in the form of an annual monitoring report, and ensure the implementation effect of the plan through dynamic monitoring^34^. Countries such as Japan, Denmark, Norway, and Sweden mainly support the green development of RBCs by utilizing clean energy^32^. Summarizing the current results of green development and urban transformation in RBCs, it can be seen that most countries or regions formulate paths for urban green development from a macro perspective, without considering the development characteristics of the city. Therefore, in the actual implementation of measures, the efficiency of green development may be low due to weak universality.

### Interval prediction method for carbon emission prediction model

Low carbon development is a common goal for the global response to energy crisis and climate change. Building carbon emission prediction models is an important means of analyzing future trends in carbon emissions^35^. However, traditional carbon peak prediction, relying heavily on point prediction methods, tends to overemphasize model outcomes and inadequately assesses associated uncertainties^36^. In contrast, interval prediction can quantify the potential uncertainty of predictions and provide decision-makers with richer information^37^. At present, interval prediction has made progress in multiple fields, such as water demand prediction^38^, electricity prediction^39^, wind energy prediction^40^, etc. In carbon emission prediction, interval prediction models are mainly divided into Markov chain models and methods based on error value probability density functions^41,42^. However, both methods have limitations: Markov chain models struggle to quantify model uncertainty, and the assumption of normally distributed error values may not align with empirical data^43^.

The Kernel Density Estimation (KDE) method, as a non-parametric estimation method, does not rely on specific assumptions regarding error distribution, but rather mines patterns in the data through analysis to obtain the probability density function of prediction errors. Therefore, integrating the KDE method into carbon emission interval prediction can more effectively quantify the uncertainty of prediction and forge a more accurate and reliable basis for formulating carbon reduction strategies for RBCs under the dual carbon background.

In summary, by synthesizing sustainable development, urban resilience, and urban transformation theories with carbon emission interval forecasting methods, RBCs can chart a green development trajectory that aligns with their unique attributes and global trends under the dual carbon context. This approach not only facilitates RBCs’ transition to a green economy and environmental enhancement but also offers valuable insights for addressing global climate change challenges.

## Overview and methods of the research area

**Fig. 1:**
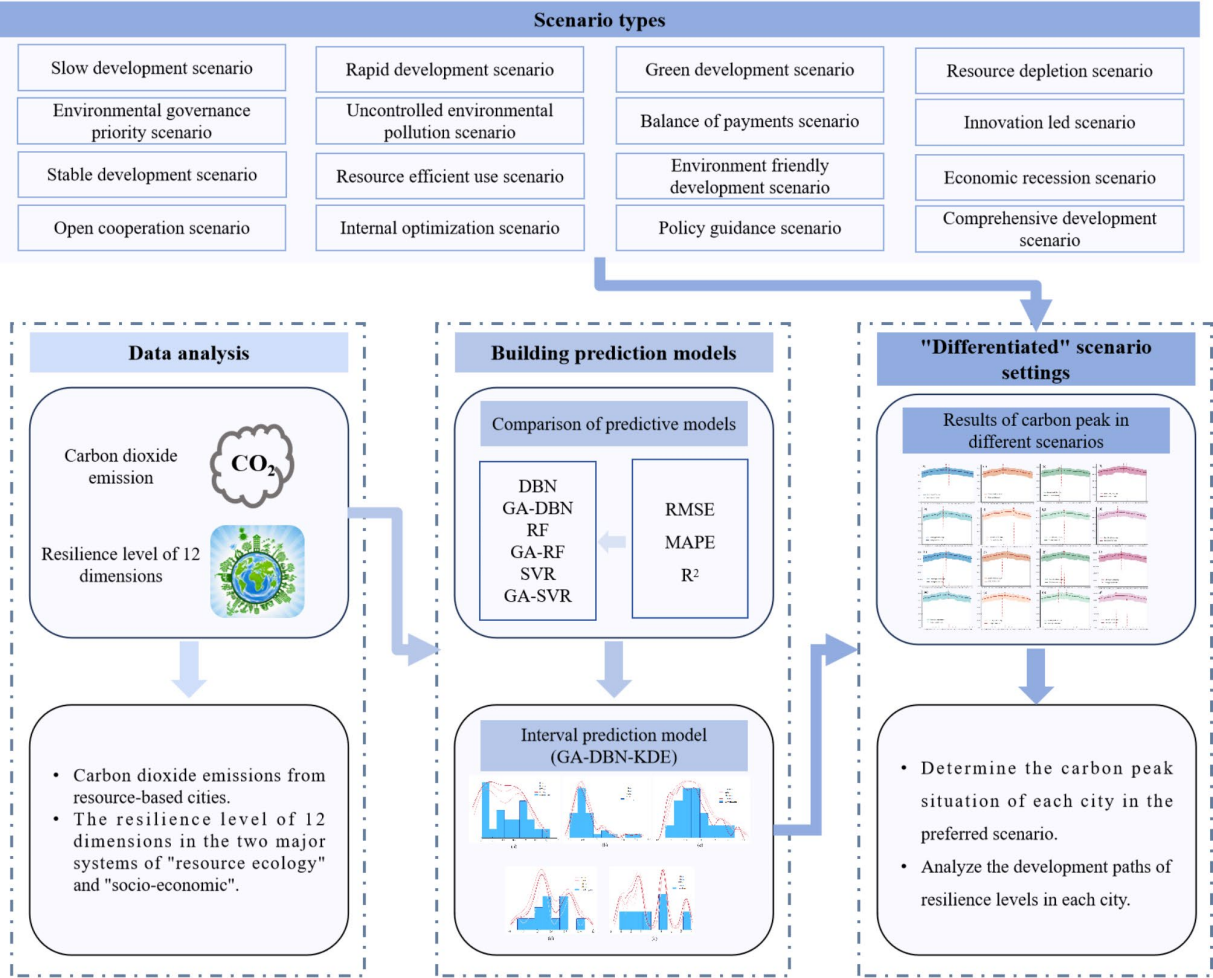
Research framework diagram.

Herein, RBCs in southwestern China were taken as the research object, urban resilience and carbon dioxide data from 2000 to 2022 was collected, and 16 scenarios of resilience development in RBCs in southwestern China were analyzed under the dual carbon background from two systems of “resource ecology” and "socio-economic". A total of 12 dimensions were involved, including water resources, land resources, mineral resources, ecological environment, environmental governance, environmental pollution, income and expenditure capacity, innovation environment, development vitality, stability, diversity, and openness. Quantify the resilience level of RBCs, and based on this, analyze 16 scenarios for RBCs to achieve low-carbon development. Exploring the scientific path of low-carbon development in RBCs under the context of “differentiation” (Fig. 1).

Step 1: Relevant data on urban resilience levels and carbon dioxide emissions of 18 RBCs in southwestern China from 2000 to 2022 was collected.

Step 2: An indicator system was built for calculating the resilience level of RBCs in Southwest China, calculate the resilience level of each city in various dimensions utilizing the optimization mutation level algorithm, and verify the stability of the optimization mutation level algorithm using the EIS index.

Step 3: A carbon emission interval prediction model was established. The resilience level of 12 dimensions in 18 cities was used as the input value of the model, and carbon emissions were used as the output value of the model. The ratio of training samples to testing samples was 8:2. The six models involved, including GA-DBN, were compared, and the optimal model was selected based on the calculation results of indicators such as RMSE, MAPE, and R^2^; A carbon emission interval prediction model was established using the KDE algorithm, and the generalization ability of the interval prediction model was evaluated using the prediction interval coverage ($PICP$) and the normalized average width of the prediction interval ($PINAW$).

Step 4: Combining existing research results and the development status of RBCs in Southwest China, 16 “differentiated” scenarios were set up, including slow development scenario, rapid development scenario, green development scenario, resource depletion scenario, environmental governance priority scenario, uncontrolled environmental pollution scenario, balance of payments scenario, innovation led scenario, stable development scenario, resource efficient use scenario, environment friendly development scenario, economic recession scenario, open cooperation scenario, internal optimization scenario, policy guidance scenario, and comprehensive development scenario;

Step 5: Based on the interval prediction model, the carbon emission point and interval prediction values of cities from 2023 to 2040 were predicted under 16 scenarios, and the suitable scenarios for low-carbon development in each city were analyzed.

### Study area

RBCs are a distinctive type of modern city, with their development closely related to the internal resource reserves of the city. This reliance on resources highlights their vulnerability, leading to challenges such as resource depletion and difficulties in urban transformation, which ultimately impact their low-carbon development. This paper selects the RBCs in southwest China as the research objects, and 18 cities in Sichuan (Dazhou, Guang’an, Guangyuan, Luzhou, Nanchong, Panzhihua, Ya’an, Zigong, Chongqing, Guizhou (Anshun, Liupanshui), Yunnan (Baoshan, Lijiang, Lincang, Puer, Qujing, Zhaotong) and Xizang (Shannan) as the research objects. The economic, cultural, and natural environment conditions of 18 cities are shown in Table 2.

**Table 2:** Overview of the study area.

City	Geographical environment	Main industries	Culture
Dazhou	The northeastern part of the Sichuan Basin is higher in the northeast and lower in the southwest	Industry	Ba culture
Guang’an	The eastern part of the Sichuan Basin has a flat terrain	Manufacturing, agriculture, and tourism industries	Bashu culture
Guangyuan	Northern Sichuan Basin	Tourism	Zhongzi fine stone culture, ancient plank road culture, Shu road culture, and three kingdoms historical culture
Luzhou	Southeast Sichuan Province	Wine and tourism industries	Famous liquor culture and ecological culture
Nanchong	Northeastern Sichuan Basin	Tourism	Three kingdoms culture, silk culture, red culture, and Jialing River culture
Panzhihua	The southernmost part of Sichuan Province, with undulating mountains	Industry	Profound intangible cultural heritage
Ya’an	Western margin of Sichuan Basin	Agriculture, Industry, and Tourism	Han culture, Tibetan culture, and Qiang culture
Zigong	Southern Sichuan Province	Industry, salt industry, and chemical industry	Salt culture, dinosaur culture, lantern festival culture
Chongqing	The eastern part of the Sichuan Basin has a relatively large terrain	Industry	Bayu culture
Anshun	Eastern edge of the Yunnan-Guizhou Plateau	Agriculture and tourism industry	Miao culture and Buyi culture
Liupanshui	Wumeng Mountain Area	Industry	Yi culture, Miao culture, and Buyi culture
Baoshan	At the border of Yunnan, Sichuan, and Myanmar provinces	Agriculture and photovoltaic industry	Yongchang culture
Lijiang	Connection between the Yunnan-Guizhou Plateau and Qinghai Tibet Plateau	Tourism	Naxi culture
Lincang	The southern extension of the Nushan Mountains in the Hengduan Mountains	Sugar Industry and Liquor Industry	Wa culture
Pu 'er	Southern Hengduan Mountains	Featured Biotechnology Industry	Tea culture and ethnic culture
Qujing	Wumeng Mountain Area, Northeast Yunnan, Central the Yunnan-Guizhou Plateau	Industry	Yi and Miao cultures
Zhaotong	North of the Yunnan-Guizhou Plateau	Industrial and service industries	Yi culture
Shannan	Gangdise Mountain to the south of Nianqing Tanggula Mountain	Agriculture	Tibetan Buddhism

### Data sources

This study reviewed and contrasted existing research, assessed key factors affecting carbon dioxide emissions in RBCs, compiled data from 2000 to 2022, and developed a scenario-based simulation framework to analyze the resilience level development of RBCs in Southwest China under the dual carbon background. The resilience of RBCs across 12 dimensions within the “resource ecology” and “social economy” systems was hereby evaluated. These dimensions mainly included water resources, land resources, mineral resources, ecological environment, environmental governance, environmental pollution, income and expenditure capacity, innovation environment, development vitality, stability, diversity, and openness (data source: during the period from 2000 to 2022, the *Statistical Yearbook of Sichuan Province*, the *Statistical Yearbook of Yunnan Province*, the *Statistical Yearbook of Chongqing*, the *Statistical Yearbook of Guizhou Province*, the *Statistical Yearbook of Xizang Autonomous Region*, etc. The resilience level was mainly calculated by optimizing the variance level algorithm). The carbon dioxide emissions of 18 RBCs in southwest China sourced from: https://www.ipe.org.cn/MapLowCarbon/LowCarbon.html?q=5. Details are shown in Table 3:

**Table 3:** indicator system for resilience level of RBCs in Southwest China.

Indicators	Indicator code	Indicator layer	References
Resource ecological resilience system			
Water resources	C1	Annual precipitation (mm)	^29^
		Total water resources (10,000 cubic meters)	
Land resources	C2	Highway mileage (kilometers)	^44^
		Total arable land resources at the end of the year (thousand hectares)	
Mineral resources	C3	Number of people in the mining industry of mineral resources (10,000 people)	^45^
		Industrial electricity consumption (10,000 kWh)	
Ecological environment	C4	Average temperature (°C)	^46^
		Air quality compliance rate	
Environmental pollution	C5	Industrial ecological energy efficiency (tons/10,000 yuan)	^22^
		Industrial pollutant emissions (tons)	
Environmental governance	C6	Industrial wastewater treatment rate	^45^
		Comprehensive utilization rate of general industrial solid waste	
Socio-economic resilience system			
Income and expenditure capacity	C7	Per capita GDP	^28^
		Local fiscal revenue	
		Per capita disposable income	^47^
Innovation environment	C8	Scientific expenditure	^4^
		Number of patent applications	
		Internal R&D expenditure	
Development vitality	C9	The GDP growth rate of the secondary industry	^48^
		The growth rate of employment in the secondary industry	
		Total retail sales of consumer goods	
Stability	C10	Basic pension insurance participation rate	^49^
		The year-end loan-to-deposit ratio of financial institutions	
Diversity	C11	Rational structure of production	^19^
		Advanced industrial structure	
Openness	C12	Actual utilization of foreign investment as a percentage of GDP	^50^
		Foreign trade volume	

### Method

This paper analyzed the specific path for RBCs in Southwest China to achieve low-carbon development under the time constraint of “carbon peak”. Two main methods were needed to process and calculate the data: (1) The optimization mutation level algorithm was used to calculate the resilience level of 12 dimensions, laying the foundation for subsequent scenario simulation analysis; (2) The interval prediction model based on GA-DBN-KDE mainly predicted the carbon emissions and “carbon peak” time of 18 RBCs from 2023 to 2040, combined with scenario simulation, aiming to provide a more reliable and scientific development path for RBCs.

#### Optimization of mutation level algorithm

The mutation series algorithm integrates mutation theory and fuzzy mathematics to generate mutation fuzzy membership functions, which are then normalized for a comprehensive assessment of the target^4^. This method evaluates both the indicators’ importance without assigning weights. Consequently, when using algorithms, the importance of indicators should be objectively considered. In this paper, the entropy weight method was employed to determine the importance, which could retain more subjectivity in the algorithm. The algorithm formula could be expressed as follows:

1 $$f(x) = \frac{1}{n + 2}x^{(n + 2)} + \frac{1}{n}ax^{n} + \frac{1}{n - 1}bx^{(n - 1)} + \cdots + \frac{1}{[n - (n - 1)]}\sigma x^{[n - (n - 1)]}$$

where $f(x)$ denotes the potential function of a state variable $x$ of a system, $n$ is the number of state variables, and $a$, $b$, …$\sigma$ are all control variables of $x$, determined by the entropy weight method, following the principle of $a > b > \cdots > \sigma$.

#### GA-DBN

**Fig. 2:**
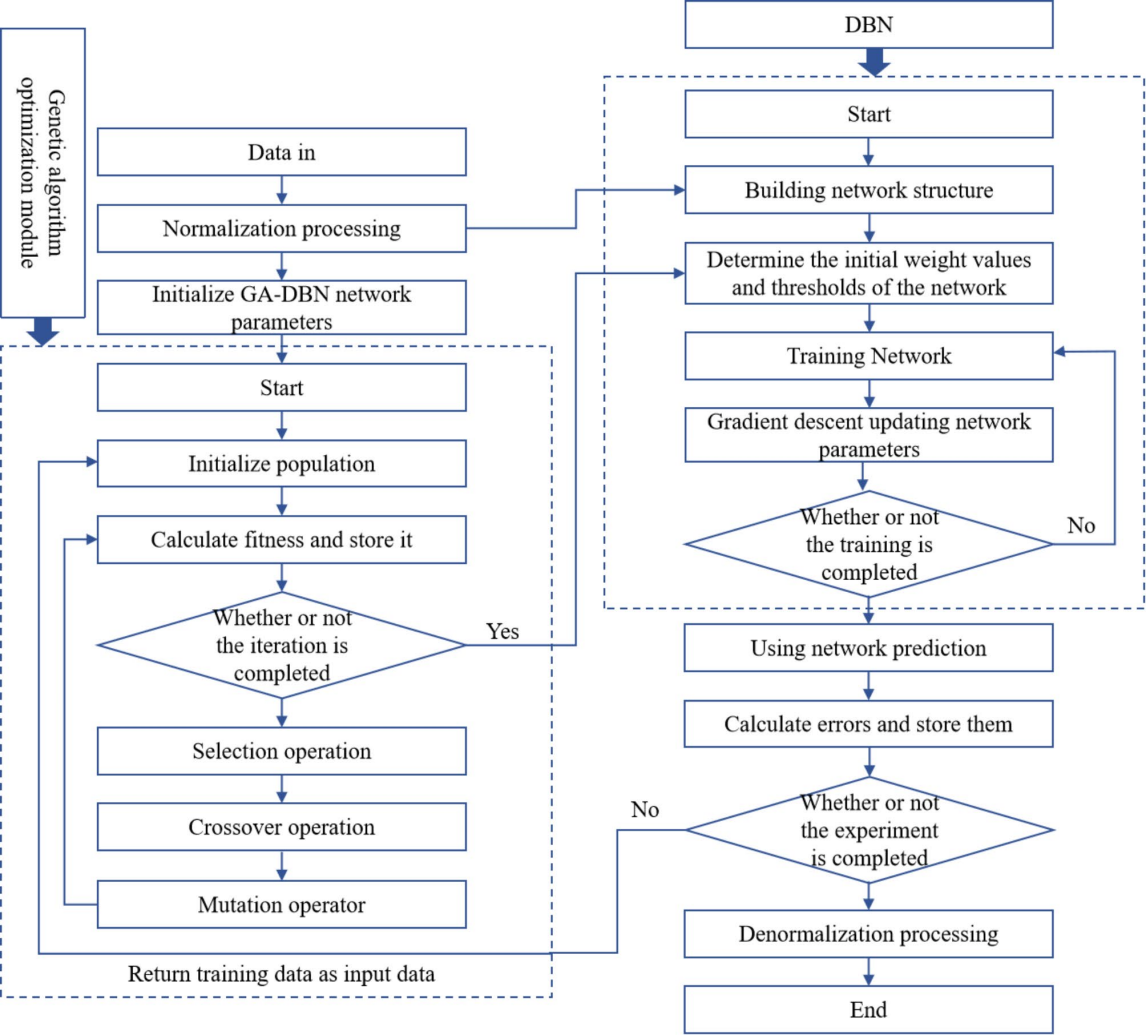
Flowchart of GA-DBN model.

Deep Belief Network (DBN) is a neural network model in deep learning methods, which is based on restricted Boltzmann machines. This model combines the characteristics of supervised and unsupervised learning, and features strong scalability and flexibility^51^. However, the training process of DBN is relatively complex, requiring careful adjustment of hyper-parameters, and may easily fall into local optima, possibly leading to poor model performance. Consequently, a parameter optimization algorithm is required to enhance the performance of DBN. Given the maturity of genetic algorithms in optimization tasks^52^, employing GA for DBN tuning is a practical approach. The specific flow is shown in Fig. 2.

#### Carbon emission interval prediction model

The simple GA-DBN prediction model can only meet the point prediction in carbon emission prediction, which leads to inaccuracy in carbon emission prediction to some extent. Kernel density estimation (KDE), as a nonparametric estimation method to obtain the probability density function without assuming a priori distribution, can obtain estimation intervals with higher accuracy and smoothness compared to other nonparametric estimation methods such as the histogram method and the nearest neighbor algorithm^53^. Among them, different bandwidth choices have different fits to the prediction error, and an adaptation optimization index $G$ is introduced to evaluate the fitting performance of KDE^38^, the smaller the index $G$, the better the fitting performance.

#### Robustness test

The robustness test aims to evaluate the explanatory stability of evaluation methods and indicators under parameter changes, ensuring that these methods and indicators can still provide consistent and reliable explanatory results even under changing conditions^54^. Herein, the results of urban resilience level were compared and analyzed using ESI and the optimization of mutation level algorithm, verifying the stability of the optimization of mutation level algorithm results and further confirming the relative objectivity of its evaluation results.

2 $$ESI = \sum\limits_{i = 1}^{10} {\varphi_{i} \times U_{i} }$$

Among them, $\varphi_{i}$ represents the weight of each indicator, $i$ is the $i$ th indicator, and $U_{i}$ is the indicator value of the indicator layer.

#### Model performance evaluation

In order to assess the accuracy of forecasting models better, four evaluation indicators including Mean Absolute Error (MAE), Root Mean Square Error (RMSE), Mean Absolute Percent Error (MAPE), and Determination Coefficient (R^2^) were used.

3 $$RMSE = \sqrt {\frac{1}{n}\sum\limits_{i = 1}^{n} {\left( {\widehat{y}_{i} - y_{i} } \right)^{2} } }$$

4 $$MAPE = \frac{100\% }{n}\sum\limits_{i = 1}^{n} {\left| {\frac{{\widehat{y}_{i} - y_{i} }}{{y_{i} }}} \right|}$$

5 $$R^{2} = 1 - \frac{{\sum\nolimits_{i = 1}^{n} {\left( {y_{i} - \widehat{y}_{i} } \right)}^{2} }}{{\sum\nolimits_{i - 1}^{n} {\left( {y_{i} - \overline{y}_{i} } \right)}^{2} }}$$

In the Equations, $n$ represents the number of samples in a sample set; and $y_{i}$ and $\widehat{y}_{i}$ represent the actual and forecast scores at the time $i$, respectively; $\overline{y}$ represents the mean of the samples taken from the test group. If the scores of RMSE, MAPE are smaller and R^2^ is larger, the prediction performance is better.

To further measure the generalization performance of the model, the prediction model performance was hereby evaluated using the $PICP$ and $PINAW$ (Du et al., 2022). The larger the $PICP$, the better the predictive model. The average width of the predicted interval was calculated using $PINAW$, and the smaller the value of $PINAW$, the better the prediction result.

## Results

### Current status of carbon dioxide emissions in RBCs in Southwest China

**Fig. 3:**
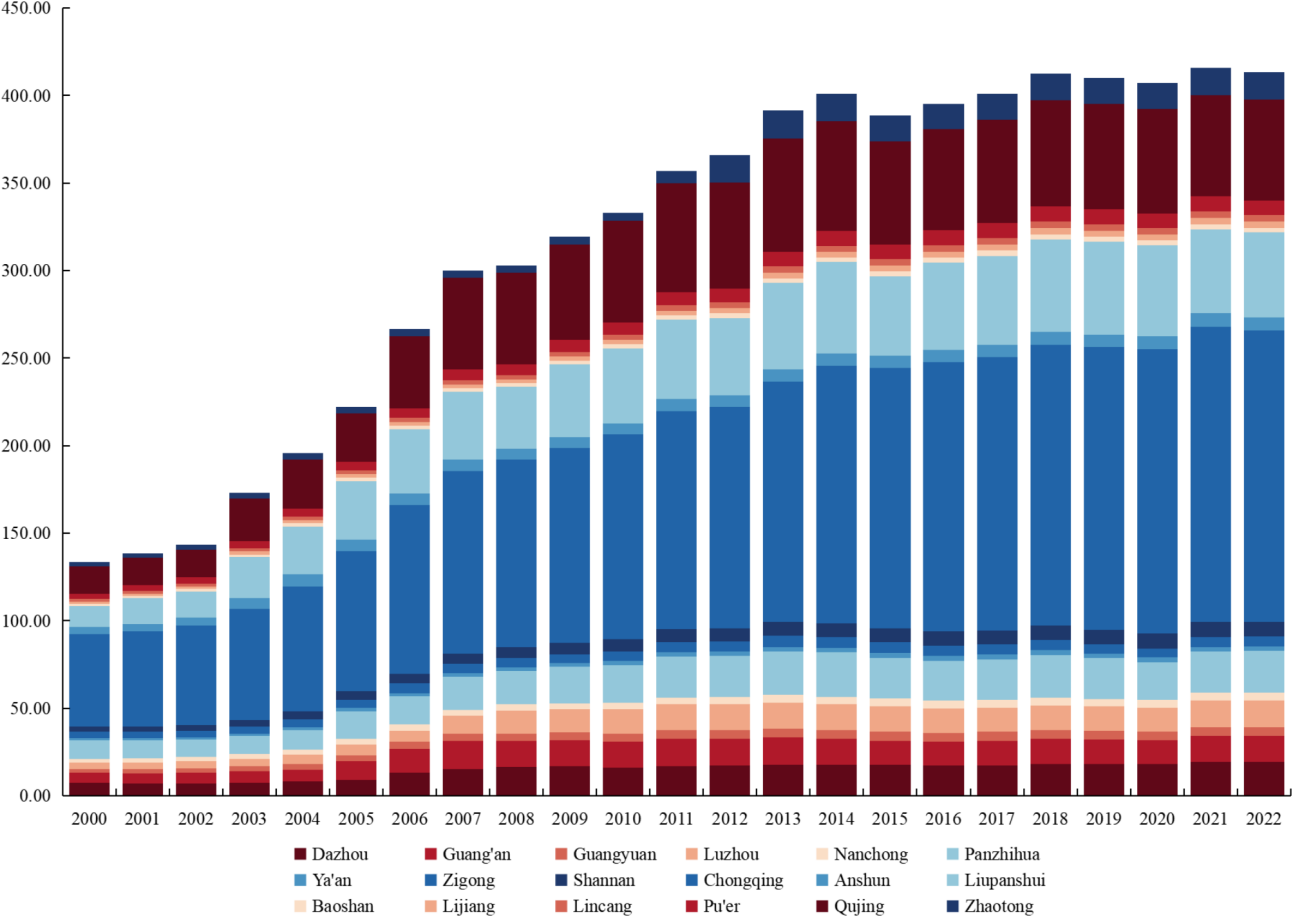
Carbon dioxide emissions of RBCs in Southwest China from 2000 to 2022.

The carbon dioxide data of 18 RBCs in Southwest China from 2000 to 2022 was collected, as shown in Fig. 3:

From Fig. 3, it could be seen that: (1) the carbon dioxide emissions in RBCs in southwestern China were basically on the rise, increasing from 133.58 million tons to 413.43 million tons; Especially during the period from 2000 to 2008, carbon emissions showed an accelerating growth trend. (2) Cities exhibited substantial variability in carbon emissions, with Chongqing leading due to its larger economic scale, population, and industrial base as a directly administered municipality. (3) There was a significant gap in the growth rate of carbon emissions, with Zhaotong having the highest annual average growth rate of 10.61% in carbon dioxide emissions, followed by Luzhou and Liupanshui with annual average growth rates of 7.52% and 7.32%, respectively. Anshun, Nanchong, and Zigong had the lowest annual average growth rate in carbon dioxide emissions, primarily due to the active development of industries in Zhaotong, Luzhou, and Liupanshui, which resulted in significant carbon emissions. However, Anshun, Nanchong, and Zigong placed greater emphasis on the development of environmental protection industries.

### Development status of resilience level of RBCs in Southwest China

The EIS index and optimization of mutation level algorithm were employed to calculate the urban resilience level, and the results are shown in Table 4. The fluctuation range of the two calculation results was approximately 0.15, indicating consistency. This suggested that the optimization of mutation level algorithm in this study was applicable in assessing urban resilience, yielding valid and rational results.

**Table 4:** Calculation results of EIS index and optimized mutation level algorithm.

Year	EIS index	Optimization mutation level algorithm
2000	0.489	0.509
2001	0.587	0.542
2002	0.656	0.561
2003	0.634	0.579
2004	0.592	0.592
2005	0.546	0.611
2006	0.627	0.602
2007	0.681	0.621
2008	0.664	0.619
2009	0.636	0.621
2010	0.630	0.625
2011	0.640	0.650
2012	0.648	0.633
2013	0.700	0.665
2014	0.598	0.648
2015	0.675	0.655
2016	0.670	0.640
2017	0.658	0.658
2018	0.665	0.650
2019	0.645	0.665
2020	0.663	0.653
2021	0.720	0.660
2022	0.643	0.658

**Fig. 4:**
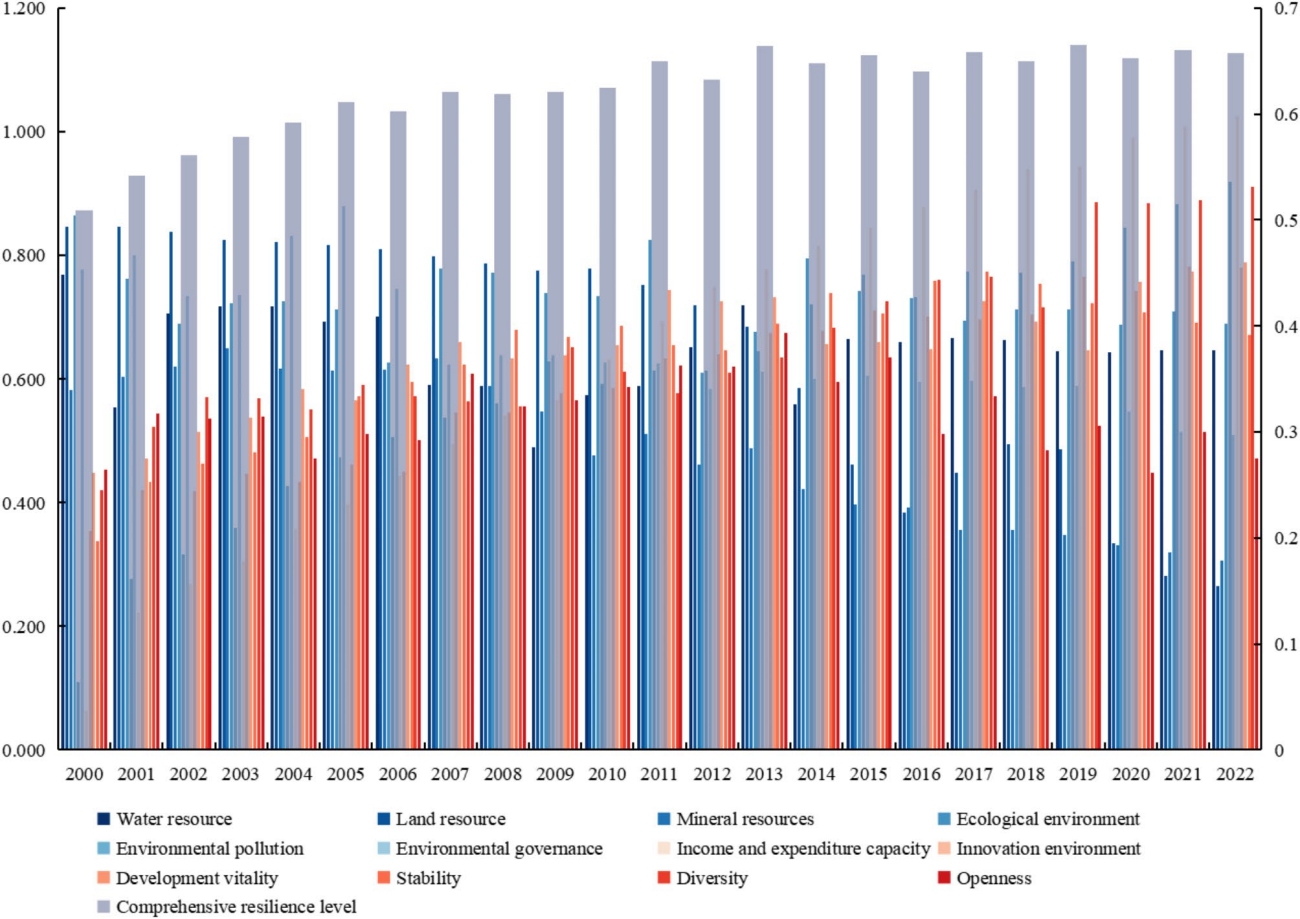
Changes in resilience levels across 12 dimensions.

From Fig. 4, it could be seen that: (1) From 2000 to 2022, the resilience level of RBCs in Southwest China showed a slow upward trend, with an average annual growth rate of 1.20%. (2) The changes in resilience levels in dimensions such as water resources and ecological environment were basically consistent, showing a "W" shaped trend, and ultimately maintaining a stable state after 2015. This indicated that in the early stages of urban development, due to extensive economic growth, there were fluctuations in water resource management and ecological environment protection. Subsequently, with the increasing awareness of environmental protection, the resilience level had rebounded and remained stable. (3) The changes in resilience levels in dimensions such as land resources, mineral resources, and environmental governance were slowly declining, indicating a decrease in the utilization efficiency of land and mineral resources and a continuous reduction in investment in environmental governance. (4) The resilience level in dimensions such as environmental pollution, income and expenditure capacity, innovative environment, development vitality, and diversity presented a clear upward trend, indicating the smooth development of cities in terms of economy and innovation.

### Prediction model construction

#### Construction of GA-DBN Model

**Fig. 5:**
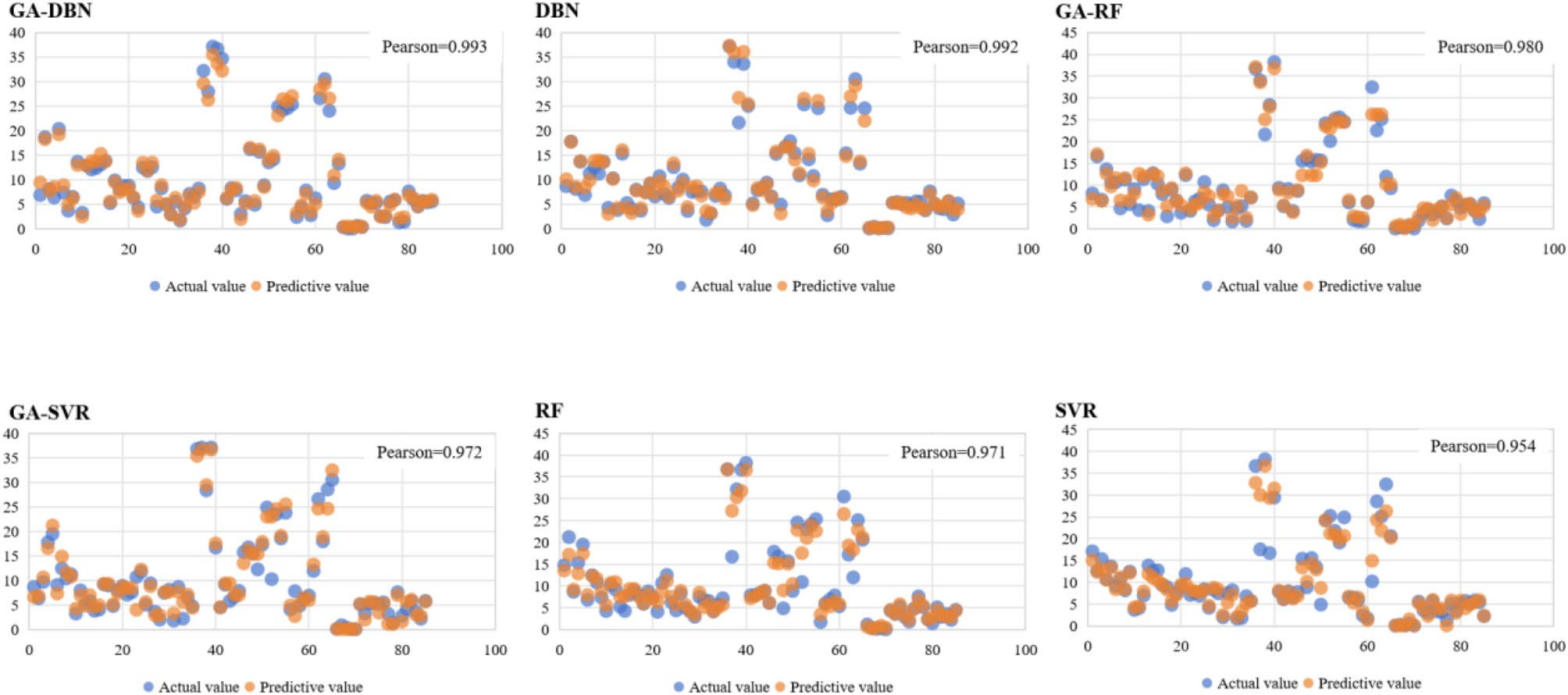
Comparison between predicted and actual values of each model.

To further explore the optimal models for predicting carbon dioxide emissions, this paper compared models such as DBN, GA-DBN, RF, GA-RF, SVR, GA-SVR, etc. The input variables of the models were resilience levels that included 12 dimensions including water resources, land resources, mineral resources, ecological environment, environmental governance, environmental pollution, income and expenditure capacity, innovation environment, development vitality, stability, diversity, and openness, with carbon emissions as the output. Among them, the sample ratio between the training set and the testing set is 8:2. Indicators such as RMSE, MAPE, and R^2^ were utilized to determine the accuracy of each model. The results are shown in Fig. 5 and Table 3:

As shown in Fig. 5, Pearson correlation coefficient analysis was performed on the predicted values and actual values of each model. The higher the correlation coefficient, the better the prediction accuracy of the model. The correlation coefficients of the six models from high to low were: GA-DBN, DBN, GA-RF, GA-SVR, RF, and SVR. Comparing the RMSE, MAPE, and R^2^ values of 6 models, as shown in Table 5, the GA-DBN model performed better in both the training and testing phases, presenting the smallest RMSE, MAPE, and R^2^ values. Therefore, the GA-DBN model was hereby chosen for carbon emission prediction.

**Table 5:** Performance evaluation results of each model.

		RMSE	MAPE	R^2^
Training set	DBN	2.856	2.156	0.979
	GA-DBN	2.152	2.012	0.984
	RF	7.698	6.235	0.916
	GA-RF	5.662	5.136	0.932
	SVR	7.089	6.198	0.909
	GA-SVR	6.396	5.987	0.957
Test set	DBN	3.213	3.198	0.959
	GA-DBN	2.986	2.955	0.967
	RF	4.128	4.213	0.839
	GA-RF	3.597	3.268	0.935
	SVR	5.636	4.698	0.855
	GA-SVR	3.295	3.659	0.942

#### Interval prediction model based on GA-DBN-KDE

The predicted values of carbon dioxide were divided into 5 levels, namely [0,5), [5,10), [10,20), [20,40), and [40, + ∞). Different KDE parameters were set to fit the probability density function of the error values. The specific bandwidth settings are shown in Table 6:

**Table 6:** KDE bandwidth settings at different levels.

	Level 1	Level 2	Level 3	Level 4	Level 5
w_1_	0.20	0.22	0.15	0.12	0.25
w_2_	0.18	0.12	0.20	0.20	0.40
w_3_	0.15	0.18	0.18	0.22	0.30
w_4_	0.22	0.15	0.12	0.18	0.35

**Fig. 6:**
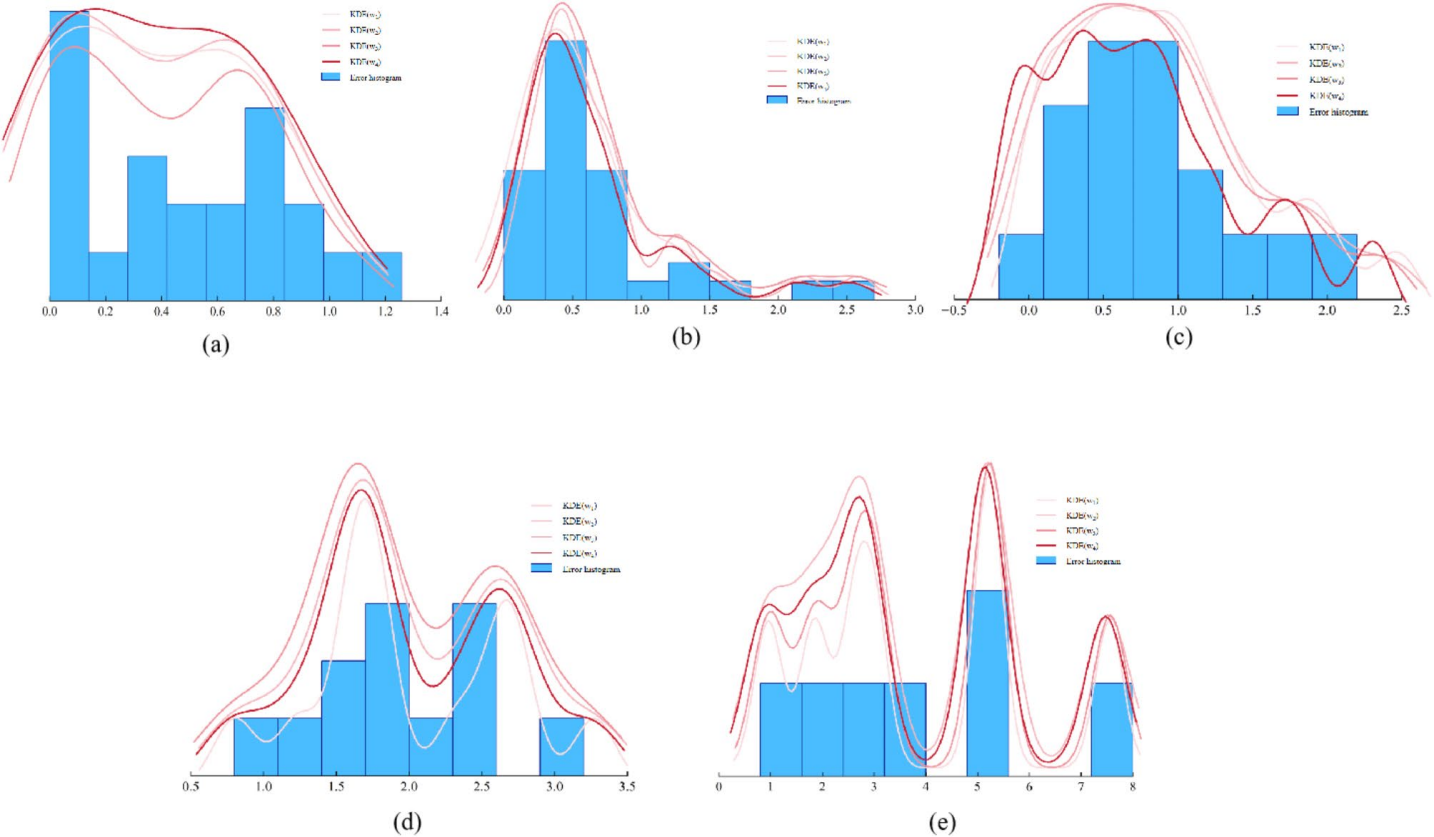
prediction error fitting curve.

As shown in Fig. 6, the fitting curve of the prediction error could to some extent characterize the distribution of the error. At this point, the $G$ index was used to further determine the optimal probability density curve. The specific results are shown in Table 7:

**Table 7:** Comparison of fitting performance.

	Adaptation optimization index $G$ value				
	Level 1	Level 2	Level 3	Level 4	Level 5
KDE(w1)	6.91332	0.05791	0.25953	**0.08847**	0.10000
KDE(w2)	6.97669	0.08285	0.37770	0.18607	**0.09912**
KDE(w3)	7.00131	0.02814	0.34555	0.16555	0.09999
KDE(w4)	**6.80266**	**0.00854**	**0.10002**	0.13035	0.09982

Bolded numbers represent the best answers in this level.

According to Table 7, the optimal bandwidths were w1 (0.22), w3 (0.15), w4 (0.12), w2 (0.12), and w2 (0.40), respectively. On this basis, the error interval of carbon dioxide was further determined using a confidence interval of 95%. The results are shown in Table 8:

**Table 8:** Confidence intervals obtained at five levels.

	Level 1	Level 2	Level 3	Level 4	Level 5
Upper and lower limit values	[-0.103,1.285]	[-1.104, 1.355]	[-0.080, 1.704]	[-1.238, 2.715]	[-1.579,7.333]
Interval width	1.388	1.459	1.784	3.953	8.913

**Fig. 7:**
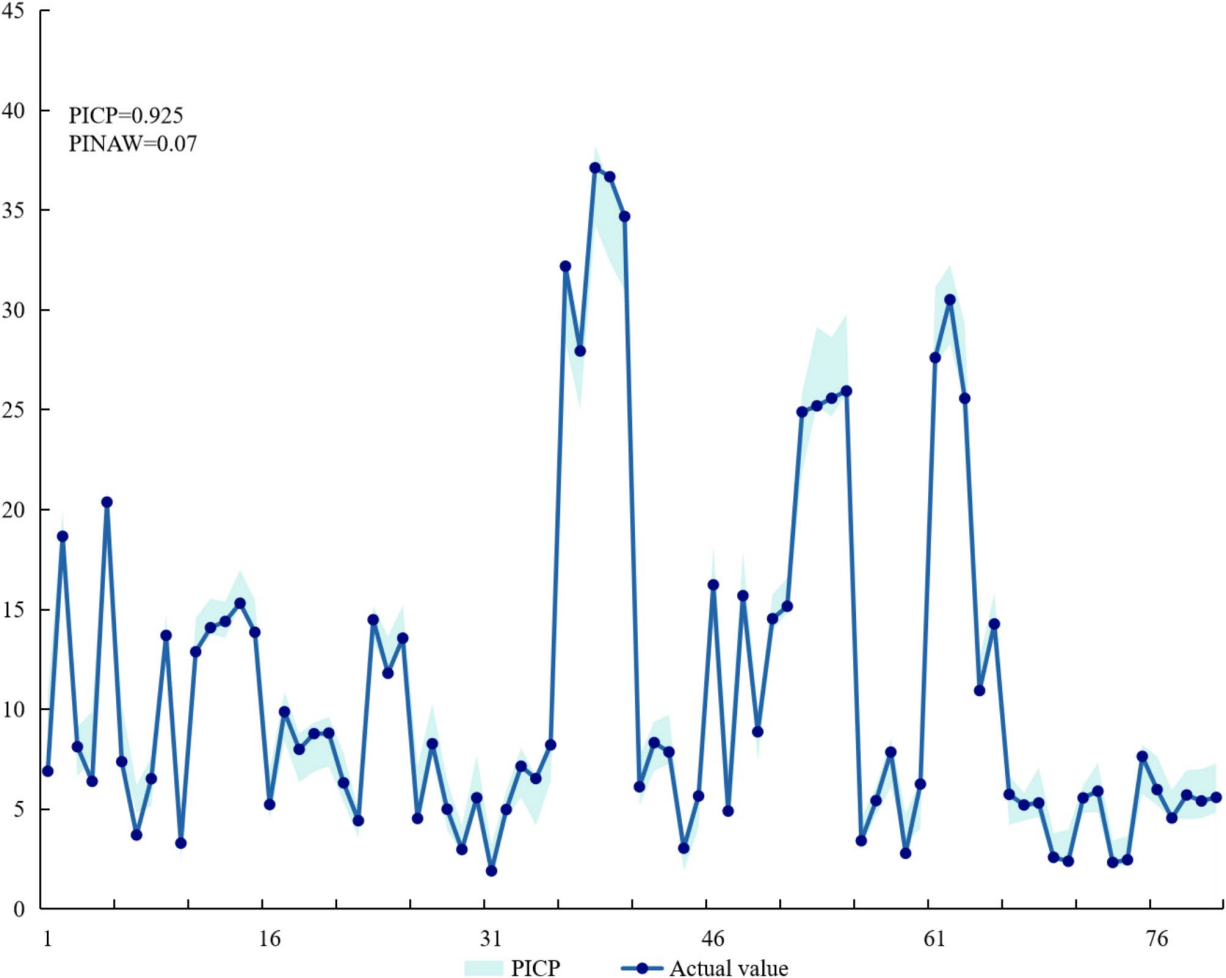
GA-DBN-KDE interval prediction chart.

In order to visually demonstrate the effectiveness of the interval prediction model, two indicators, *PICP* and *PINAW,* were hereby selected to evaluate the interval prediction results. As indicated by Fig. 7, the *PICP* was 0.925, and the *PINAW* was 0.07, indicating that the interval prediction model could accurately reflect the prediction of carbon dioxide.

### Scenario setting

#### Setting the Growth Rate of Influencing Factors

Drawing on the development plan, long-term goals, and policy measures of RBCs in Southwest China, and fully considering the economic development process of developed countries and regions, this paper divided the resilience level of 12 dimensions into 3 development rates: high, medium, and low. It was assumed that the resilience level of 18 RBCs in Southwestern China was developing at the stated rate. Based on the specific situation of the 12 dimensions of RBCs from 2000 to 2022, the 12 dimensions were fitted into logarithmic and exponential functions: (1) slowly decreasing logarithmic functions: resilience levels of water resources, resilience levels of ecological environment, and resilience levels of environmental governance; (2) slowly increasing logarithmic functions: resilience levels of environmental pollution, resilience levels of income and expenditure capacity, resilience levels of innovation environmental, resilience levels of development vitality, resilience levels of stability, and resilience levels of openness; (3) slowly declining exponential functions: resilience levels of land resources and resilience levels of mineral resources; and (4) slow rising exponential functions: resilience levels of diversity. The specific parameter settings are shown in Table 9:

**Table 9:** Development rates of impact factors.

Impact factor	Rate of influence	2021–2025	2025–2030	2030–2040	Impact factor	Rate of influence	2021–2025	2025–2030	2030–2040
C1	High	-0.05%	-0.04%	-0.02%	C7	High	1.39%	1.16%	0.94%
	Medium	-0.10%	-0.09%	-0.07%		Medium	1.34%	1.11%	0.89%
	Low	-0.15%	-0.14%	-0.12%		Low	1.29%	1.06%	0.84%
C2	High	-1.54%	-1.24%	-0.90%	C8	High	0.65%	0.55%	0.45%
	Medium	-1.59%	-1.29%	-0.95%		Medium	0.60%	0.50%	0.40%
	Low	-1.64%	-1.34%	-0.10%		Low	0.55%	0.45%	0.35%
C3	High	-1.05%	-0.87%	-0.66%	C9	High	0.42%	0.36%	0.29%
	Medium	-1.10%	-0.92%	-0.71%		Medium	0.37%	0.31%	0.24%
	Low	-1.15%	-0.97%	-0.76%		Low	0.32%	0.26%	0.19%
C4	High	-0.06%	-0.04%	-0.02%	C10	High	0.65%	0.55%	0.45%
	Medium	-0.11%	-0.09%	-0.07%		Medium	0.60%	0.50%	0.40%
	Low	-0.16%	-0.14%	-0.12%		Low	0.55%	0.45%	0.35%
C5	High	1.03%	0.86%	0.69%	C11	High	2.37%	2.70%	3.28%
	Medium	0.98%	0.81%	0.64%		Medium	2.32%	2.65%	3.23%
	Low	0.93%	0.76%	0.59%		Low	2.27%	2.60%	3.18%
C6	High	-0.34%	-0.27%	-0.21%	C12	High	0.15%	0.14%	0.12%
	Medium	-0.39%	-0.32%	-0.26%		Medium	0.10%	0.09%	0.07%
	Low	-0.44%	-0.37%	-0.31%		Low	0.05%	0.04%	0.02%

#### Scenario type setting

This paper has 12 indicators that can simulate 531441 scenarios. However, in actual scenario simulations, using too many scenarios may lead to unclear analysis^55^ Therefore, in the context of “dual carbon”, this paper fully considered the development status of RBCs in Southwest China, explored the carbon peak situation of each city, and studied the low-carbon development path of each city. Scenario analysis was carried out to predict the possible impact of different development models on carbon emissions. The growth rates of corresponding influencing factors under different scenarios are shown in Table 10, and the specific scenario descriptions are as follows:

**Table 10:** Scenario setting parameters table.

Type	C1	C2	C3	C4	C5	C6	C7	C8	C9	C10	C11	C12
Scenario 1	L	L	L	L	L	L	L	L	L	L	L	L
Scenario 2	H	H	H	H	H	H	H	H	H	H	H	H
Scenario 3	L	L	L	M	L	L	M	H	H	H	M	H
Scenario 4	H	H	H	M	H	H	L	M	L	L	M	L
Scenario 5	L	L	L	M	L	L	L	M	M	M	H	H
Scenario 6	H	H	H	L	H	H	L	M	L	L	L	L
Scenario 7	M	M	M	M	L	M	M	H	M	M	M	M
Scenario 8	L	L	L	M	L	M	M	H	H	M	H	H
Scenario 9	L	L	L	H	L	L	M	H	M	H	H	M
Scenario 10	L	L	L	M	H	M	H	M	M	M	H	H
Scenario 11	L	L	L	H	H	L	H	M	H	H	H	H
Scenario 12	L	L	L	M	H	H	L	L	L	L	L	L
Scenario 13	L	L	L	M	L	M	H	M	H	M	H	H
Scenario 14	L	L	L	M	L	M	M	M	M	H	H	M
Scenario 15	L	L	L	H	L	L	M	M	M	M	H	H
Scenario 16	L	L	L	H	L	L	M	M	H	M	H	H

Note: L is low; H is high; M is medium.

Scenario 1: Slow development scenario. All influencing factors maintained a “low” development rate, characterized by minimal resource consumption and weak environmental pollution. Environmental governance was minimal, and the economy was gradually improving. Urban openness and development momentum were reduced.

Scenario 2: Rapid development scenario. All influencing factors maintained a “high” development rate, marked by intense resource consumption and escalating environmental pollution. Despite increased environmental governance, it struggled to match the pace of pollution. The economy grew rapidly, with robust urban openness and development momentum.

Scenario 3: Green development scenario. Attention was paid to the sustainable utilization of water resources, land resources, and mineral resources. Environmental pollution was effectively controlled with robust environmental governance efforts. Economic development was aligned with environmental protection, fostering the growth of innovative green industries. The city maintained its openness and dynamism, with an emphasis on stability.

Scenario 4: Resource depletion scenario. Water resources, land resources, and mineral resources were rapidly depleted. Environmental pollution was severe, and environmental governance failed to reverse the damage. The economy was in decline, and urban development momentum was lost. The city’s openness and stability were diminishing.

Scenario 5: Environmental governance priority scenario. Investment in environmental governance was increased to curb pollution. Resource consumption was partially managed, yet economic growth had decelerated. Urban openness and dynamism were preserved, with a focus on diverse, inclusive development.

Scenario 6: Uncontrolled environmental pollution scenario. Environmental pollution was rapidly intensifying, and environmental governance could not respond effectively. The acceleration of resource consumption seriously affected economic development. The openness and development momentum of cities had significantly declined. Social stability was threatened.

Scenario 7: Balance of payments Scenario. A balance was maintained between economic development, resource consumption, and environmental governance. Environmental pollution was effectively controlled, yet still leaving room for improvement. The city had moderate openness and development vitality, and good stability. The innovation environment received certain support.

Scenario 8: Innovation led scenario. The innovative environment became the core driving force for development. High resource utilization efficiency and effective control of environmental pollution were achieved. The economy was developing rapidly, and cities had strong openness and development vitality, with emphasis on diversity and openness to promote inclusive growth.

Scenario 9: Stable development scenario. Economic development maintained stable growth, and resource consumption and environmental governance were controllable. Environmental pollution was effectively controlled, and environmental quality continued to improve. The city maintained a certain level of openness and development vitality, presenting strong stability. The industrial structure was reasonable, and the innovation environment was supported.

Scenario 10: Resource efficient use scenario. Emphasis was placed on the efficient utilization of water resources, land resources, and mineral resources. Environmental pollution was effectively controlled, and the intensity of environmental governance was moderate. Economic development was resource-efficient and sustainable. Urban openness and vitality were sustained, with a focus on diversity and inclusiveness.

Scenario 11: Environment friendly development scenario. Economic development was based on environmental protection, and green development and circular economy were emphasized. Resource consumption was effectively controlled, and environmental pollution was significantly reduced. Environmental governance was robust, leading to ongoing improvements in environmental quality. The city exhibited strong openness and development vitality, with an emphasis on stability and diversity.

Scenario 12: Economic recession scenario. Economic development had fallen into recession, with reduced resource consumption yet low utilization efficiency. Environmental pollution was worsening, and there was insufficient investment in environmental governance. The city’s openness and development momentum had significantly waned, with stability being compromised. The industrial structure was deemed irrational, and the innovation environment was deteriorating.

Scenario 13: Open cooperation scenario. The city prioritized openness and cooperation, attracting external resources and investment. It achieved high resource efficiency and effectively managed environmental pollution. The economy was rapidly developing, and the city maintained strong openness and vitality, with a focus on diversity and inclusivity, fostering international cooperation and exchange.

Scenario 14: Internal optimization scenario. Attention was paid to the optimization and upgrading of the internal structure of the city, and the efficiency of resource utilization was improved. Environmental pollution was effectively controlled, presenting moderate intensity of environmental governance. The economy sustained steady growth, with urban development exhibiting moderate openness and vitality. There was an emphasis on diversity and stability, fostering internal harmony and development.

Scenario 15: Policy guidance scenario. The government guided the efficient utilization of resources and environmental protection through policies. Environmental pollution was effectively controlled, and efforts to improve the environment had been intensified. Economic development was centered around policy guidance, with an emphasis on sustainable development. A certain level of urban openness and development vitality were maintained, with a focus on diversity and inclusiveness.

Scenario 16: Comprehensive development scenario. Various factors such as economic development, resource utilization, environmental protection, and social governance were taken into account. Resource consumption was effectively controlled, and environmental pollution was significantly reduced. Environmental governance was robust, leading to ongoing environmental quality improvements. The economy grew rapidly and steadily, with urban development characterized by strong openness and vitality. There was an emphasis on diversity and inclusivity, fostering comprehensive, balanced, and sustainable development.

#### Scenario analysis of carbon emissions in RBCs in Southwest China

**Fig. 8:**
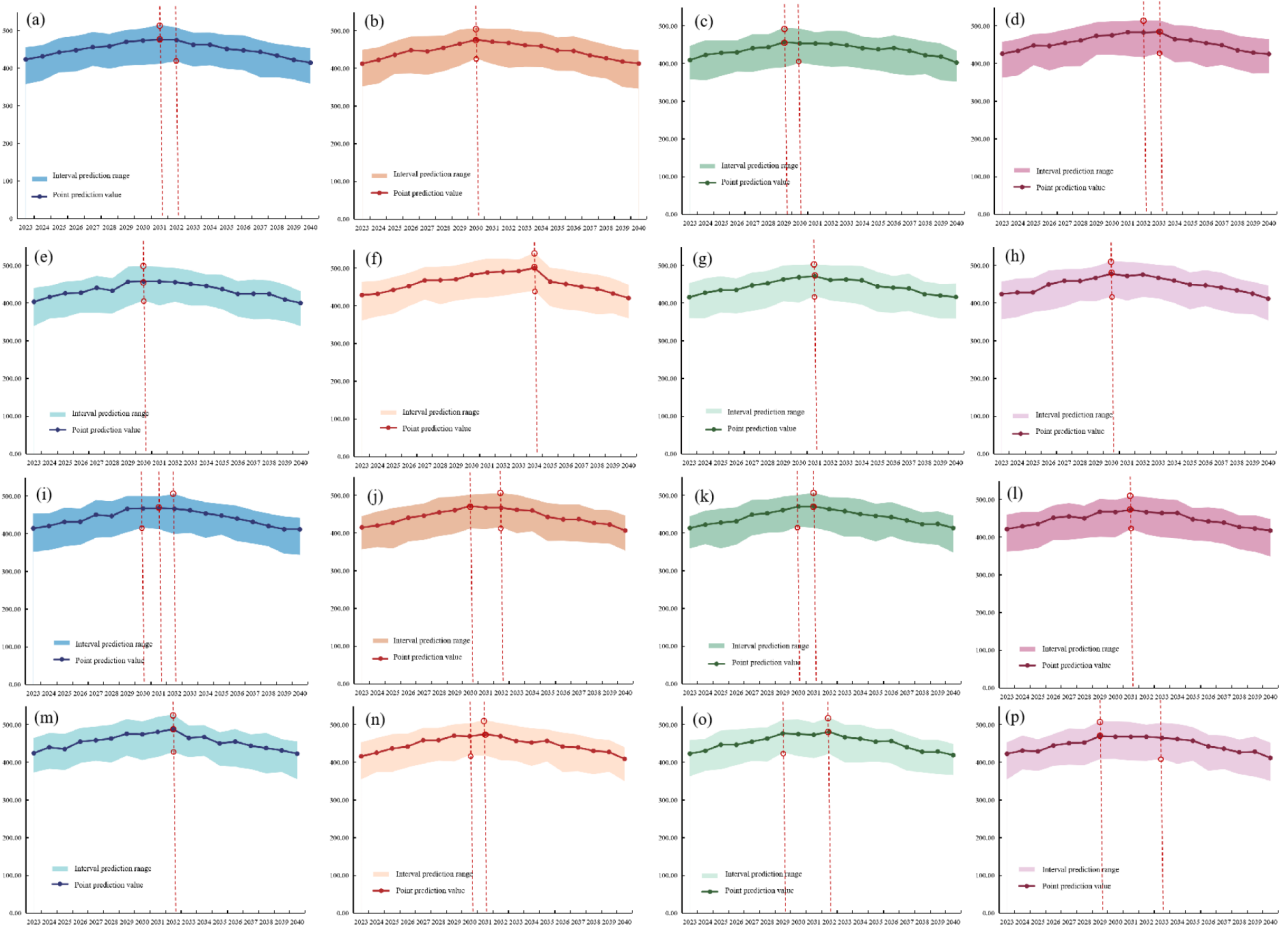
Prediction results of carbon emission intervals under different scenarios.

Based on the resilience levels of 12 dimensions, the GA-DBN-KDE model was employed to predict the carbon emissions data of 18 RBCs in Southwestern China under 16 scenarios from 2023 to 2040. The results are shown in Table 11 and Fig. 8. Southwest China was projected to reach its carbon peak between 2029 and 2034, with a peak of 402.13–540.93 million tons. Based on the projected carbon peak timeframes, the 16 scenarios were categorized into three groups. The first group, with certain peak by 2030, included scenarios 2, 3, 5, and 8. The second group, possibly peaking by 2030, encompassed scenarios 9, 10, 11, 14, 15, and 16. The third group, unlikely to peak by 2030, consisted of scenarios 1, 4, 6, 7, 12, and 13.

**Table 11:** Prediction results of carbon dioxide emissions under different scenarios.

Type	Peak carbon by 2030	Peak carbon time	Point projected peak carbon emissions (million tons)	Range of carbon emission bands (million tons)
Scenario 1	No	[2031 ~ 2032]	476.20	[418.23 ~ 516.27]
Scenario 2	Yes	[2030]	476.40	[426.46 ~ 507.42]
Scenario 3	Yes	[2029 ~ 2030]	457.09	[402.13 ~ 496.00]
Scenario 4	No	[2032 ~ 2033]	484.08	[427.06 ~ 515.54]
Scenario 5	Yes	[2030]	458.02	[407.04 ~ 498.07]
Scenario 6	No	[2034]	500.94	[440.89 ~ 540.93]
Scenario 7	No	[2031]	471.47	[419.45 ~ 503.37]
Scenario 8	Yes	[2030]	478.19	[417.18 ~ 512.14]
Scenario 9	Possible	[2030 ~ 2032]	468.61	[416.67 ~ 505.73]
Scenario 10	Possible	[2030 ~ 2032]	472.47	[416.45 ~ 506.48]
Scenario 11	Possible	[2030 ~ 2031]	471.08	[417.67 ~ 507.35]
Scenario 12	No	[2031]	473.33	[422.35 ~ 509.29]
Scenario 13	No	[2032]	488.30	[427.39 ~ 527.27]
Scenario 14	Possible	[2030 ~ 2031]	475.00	[419.45 ~ 512.89]
Scenario 15	Possible	[2029 ~ 2032]	481.15	[426.23 ~ 519.22]
Scenario 16	Possible	[2029 ~ 2033]	470.13	[410.40 ~ 510.00]

In the first category, the predicted peak carbon emissions for the four scenarios were 476.40, 457.09, 458.02, and 478.19 million tons, respectively. The corresponding peak carbon emissions ranged from 426.46 to 507.42, 402.13 to 496.00, 407.04 to 498.07, and 417.18 to 512.14 million tons. According to the scenario settings, all four scenarios emphasized the innovation environment, development vitality, stability, diversity, and openness of the city. The government adopted innovative technological means to improve resource utilization, optimize industrial structure, reduce environmental pollution, and thus reduce investment in environmental governance. It also emphasized the development of ecological environment and environmental governance, fundamentally reducing carbon emissions and achieving “carbon peak” as soon as possible.

In the second category, the peak carbon emissions predicted for the six scenarios were 468.61, 472.47, 471.08, 475.00, 481.15, and 470.13 million tons, respectively. The corresponding peak carbon emissions ranged from 416.67 to 505.73, 416.45 to 506.48, 417.67 to 507.35, 419.45 to 512.89, 426.23 to 519.22, and 410.40 to 510.00 million tons. According to the scenario settings, all six scenarios emphasized the importance of resource utilization and ecological environment, indicating that carbon emissions could be effectively controlled by continuously improving resource utilization efficiency and optimizing resource utilization technologies.

In the third category, the peak carbon emissions predicted for the six scenarios were 476.20 million tons, 484.08 million tons, 500.94 million tons, 471.47 million tons, 473.33 million tons, and 488.30 million tons, respectively. The corresponding peak carbon emissions ranged from 418.23 to 516.27 million tons, 427.06 to 515.54 million tons, 440.89 to 540.93 million tons, 419.45 to 503.37 million tons, 422.35 to 509.29 million tons, and 427.39 to 527.27 million tons. Compared to the other two categories, in the third scenario, there was insufficient emphasis on urban stability, indicating that achieving carbon peak as soon as possible required a relatively stable urban development foundation to provide stable development space for carbon reduction.

Variations in economic development models and other factors across cities resulted in a limited universality of individual development scenarios when applied at the city level. In response to this, under the goal of “dual carbon”, this paper comprehensively considered the first category and second category scenarios and selected the optimal development scenario for 18 RBCs in Southwest China (Table 12).

**Table 12:** Optimal development scenarios for RBCs.

Type	City
Scenario 2	Shannan
Scenario 3	Dazhou and Zigong
Scenario 5	Panzhihua
Scenario 8	Lijiang, Nanchong, Guangyuan
Scenario 9	Luzhou and Lincang
Scenario 10	Liupanshui and Baoshan
Scenario 11	Ya’an, Guang’an
Scenario 14	Anshun and Zhaotong
Scenario 15	Chongqing
Scenario 16	Pu’er, Qujing

## Conclusion

### Main conclusion

RBCs play a major role in the development of various countries, and RBCs in southwestern China have the characteristics of fragile ecological environment and slow economic development. With a full understanding of the “dual carbon” goal, RBCs in southwestern China were hereby adopted as the research object, relevant data was collected from 18 cities from 2000 to 2022, and the GA-DBN-KDE algorithm was employed to predict the carbon emissions of RBCs in southwestern China, exploring the scientific path of low-carbon development of RBCs from the perspective of urban resilience in the context of “differentiation” The specific conclusion is as follows:

1. From 2000 to 2022, RBCs in Southwestern China showed an overall upward trend in carbon dioxide emissions, rising from 133.58 million tons to 413.43 million tons. Cities exhibited substantial differences in emissions, with Chongqing recording the highest and Zhaotong showing the steepest growth rate, contrasted with slower growth in Anshun and other regions. Meanwhile, the overall resilience level of these cities was increasing slowly, with an average annual growth rate of 1.20%.
2. From the prediction of carbon emissions in RBCs in southwestern China, the GA-DBN model was compared with five other models. Utilizing 80% of the data for training and 20% for validation, the GA-DBN model demonstrated superior performance in both training and testing, as evidenced by metrics such as RMSE, MAPE, and R^2^. A GA-DBN-KDE interval prediction model was constructed using the KDE algorithm, and the *PICP* and (0.925) *PINAW* (0.07) were calculated separately. The results showed that the interval prediction model exhibited a strong generalization effect, effectively capturing both the predicted values and the fluctuations in urban carbon emissions.
3. The interval prediction model was employed to predict the carbon emissions of RBCs in Southwest China from 2023 to 2040 under the “differentiated” scenario. The results showed that under the 16 scenarios, the “peak carbon” time ranged basically between 2029 and 2034, while the peak carbon emissions were between 402.13–540.93 million tons. Scenarios 2, 3, 5, 8, 9, 10, 11, 14, 15, and 16 might reach their peak in 2030. This indicated that RBCs in southwestern China could achieve a “carbon peak” by 2030 with an effective resilience development model. Taking into account these 10 scenarios, reasonable low-carbon development paths were developed for 18 cities.

### Policy recommendations

In view of the above conclusions on the resilience level calculation, carbon emission prediction and scenario analysis of RBCs in Southwest China, some policy suggestions were put forward for each city:

1. For cities applying Scenario 2: Shannan. Given its relatively underdeveloped status with agriculture as the predominant industry, Shannan should focus on bolstering the resilience of its “resource ecology” and “social economy” in future development efforts. Under the guidance of "several opinions on accelerating the construction of a resource-saving and environment-friendly society", the efficiency of resource utilization should be improved through innovative scientific and technological development, and efforts should also be made to vigorously develop other industries while ensuring agricultural development. To enhance inter-city collaboration and communication, Shannan, situated in Tibet with its vast land and sparse population, must leverage urban relevance, foster the exchange of specialty products, and stimulate tourism development.
2. For cities applying Scenario 3: Dazhou and Zigong. Dazhou and Zigong, as industrial RBCs in Sichuan, should diligently implement the directives from the CPC Central Committee and the State Council on advancing the comprehensive development of a beautiful China. They must prioritize the sustainable use of water, land, and mineral resources in their future developmental strategies. Meanwhile, efforts should also be made to optimize the industrial structure, promote the green and low-carbon transformation of traditional industries, and encourage the development of green and low-carbon industries such as energy-saving and environmental protection equipment manufacturing and clean energy.
3. For cities applying Scenario 5: Panzhihua. Panzhihua, primarily an industrial city with significant vanadium-titanium iron ore reserves, should boost environmental treatment investments, ensuring such expenditures constitute a dedicated portion of the financial budget with annual increments. The focus should be on the prevention and control of air, water, and soil pollution, as well as ecological restoration. Priority should be given to the implementation of a number of key environmental protection projects, such as comprehensive utilization of solid waste, industrial wastewater treatment, urban black and odorous water treatment, etc., to ensure the effectiveness of the projects.
4. For cities applying Scenario 8: Lijiang, Nanchong, Guangyuan. Currently focusing on tourism and reducing the secondary industry’s share, the three cities should foster economic growth and urban openness, enhance cooperation and exchanges with neighboring regions, and drive regional coordinated development in subsequent development efforts. It is also advisable to emphasize diversity and openness, promote inclusive growth, respect and protect cultural diversity, and promote cultural exchanges and integration.
5. For cities applying Scenario 9: Luzhou and Lincang. At present, the two cities are primarily engaged in the wine industry, sugarcane industry and tourism, yielding good economic returns and certain urban vitality. Therefore, in subsequent development, these cities should bolster leadership to oversee stable development efforts, ensuring the coordination and implementation of various policies and measures. Detailed implementation plans and supporting policies should be formulated to ensure the effective implementation of policies and measures.
6. For cities applying Scenario 10: Liupanshui and Baoshan. The two cities are currently rich in industrial development, but lack the guidance of leading industries. In their ongoing development, the two cities should rigorously execute resource utilization plans, enhance the resource utilization structure, and preserve resource protection limits. They should also develop a leading industry centered on clean energy, such as the computer industry.
7. For cities applying Scenario 11: Ya’an and Guang’an. The two cities are involved in tourism, manufacturing, and agriculture, and the two cities are rich in cultural heritage. Therefore, in the follow-up development process, the two cities should optimize the business environment, continue to promote the reform of "release, management, and service", simplify the administrative examination and approval process, and improve the efficiency of government services. Meanwhile, efforts should also be made to strengthen the protection of intellectual property rights and market supervision, and create a good environment for innovation and entrepreneurship.
8. For cities applying Scenario 14: Anshun and Zhaotong. These two cities are RBCs in Yunnan, and they are rich in national culture. Therefore, in the subsequent development process, the two cities should encourage and support the transformation and upgrading of traditional industries, vigorously develop emerging industries and high-tech industries, and enhance industrial added value and market competitiveness. Anshun should speed up the construction of Guizhou Mountainous characteristic navigation industry system, and Zhaotong should vigorously develop plateau characteristic agricultural products industry to promote industrial agglomeration and cluster development.
9. For cities applying Scenario 15: Chongqing. As a municipality directly under the central government, Chongqing has a well-developed industrial structure and economy. In order to achieve the “carbon peak” as soon as possible, the city should adhere to policies supporting market entities in the Chengdu-Chongqing economic zone and Chongqing’s "waste-free city" quality enhancement plan, as detailed in the Exposure Draft and associated regulations, to ensure stable development.
10. For cities applying Scenario 16: Pu’er and Qujing. The two cities, with their diverse industries, stable development, and rich cultural heritage, should establish and refine their environmental supervision systems. They must enhance environmental monitoring, early warning, and emergency response capabilities and enforce strict penalties for environmental violations in subsequent development. Attention should paid to vulnerable groups, promoting the equalization of public services such as education, health care, and employment, and enhancing social stability.

## Discussion

This paper focused on analyzing the universality of carbon peak pathways in RBCs in southwestern China from the perspective of urban resilience level. Taking RBCs in Southwest China as the research object, this paper collected the relevant data from 2000 to 2022 from the perspective of time series, constructed the interval prediction model of GA-DBN-KDE, and explored the scientific path of low-carbon development of RBCs in the context of “differentiation”. However, improvements could be made in the following aspects:

1. While constructing the resilience level calculation index system in this paper, existing research should be thoroughly reviewed. However, due to data availability constraints, the selection of specific indicators included some subjective elements, which should be refined in future studies^56^.
2. When conducting “differentiated” scenario simulations in this paper, the specific characteristics of the 16 simulated scenarios have fully considered the characteristics of RBCs in southwestern China and existing research content. However, subsequent research must account for the dynamic and complex nature of urban development and implement long-term monitoring of urban “carbon peak” simulations^57^.

## Data Availability

The data will be provided by the corresponding author upon request.
